# Management of adhesive small bowel obstruction: the results of a large retrospective study

**DOI:** 10.1007/s00384-023-04512-8

**Published:** 2023-09-05

**Authors:** E. Maienza, G. Godiris-Petit, S. Noullet, F. Menegaux, N. Chereau

**Affiliations:** Department of General and Endocrine Surgery, Hospital Pitié Salpêtrière, APHP, Sorbonne University Paris, 47-83 Boulevard de l’Hôpital, 75651 Paris Cedex 13, France

**Keywords:** Small bowel obstruction, Water-soluble contrast agent, Diagnostic protocol, Oral gastrografin

## Abstract

**Background:**

Postoperative adhesive small bowel obstruction (SBO) is a frequent cause of hospital admission in a surgical department. Emergency surgery is needed in a majority of patients with bowel ischemia or peritonitis; most adhesive SBO can be managed nonoperatively. Many studies have investigated benefits of using oral water-soluble contrast to manage adhesive SBO. Treatment recommendations are still controversial.

**Methods:**

We conducted an observational retrospective monocentric study to test our protocol of management of SBO using Gastrografin^®^, enrolling 661 patients from January 2008 to December 2021. An emergency surgery was performed in patients with abdominal tenderness, peritonitis, hemodynamic instability, major acute abdominal pain despite gastric decompression, or CT scan findings of small bowel ischemia. Nonoperative management was proposed to patients who did not need emergency surgery. A gastric decompression with a nasogastric tube was immediately performed in the emergency room for four hours, then the nasogastric tube was clamped and 100 ml of nondiluted oral Gastrografin^®^ was administered. The nasogastric tube remained clamped for eight hours and an abdominal plain radiograph was taken after that period. Emergency surgery was then performed in patients who had persistent abdominal pain, onset of abdominal tenderness or vomiting during the clamping test, or if the abdominal plain radiograph did not show contrast product in the colon or the rectum. In other cases, the nasogastric tube was removed and a progressive refeeding was introduced, starting with liquid diet.

**Results:**

Seventy-eight percent of patients with SBO were managed nonoperatively, including 183 (36.0%) who finally required surgery. Delayed surgery showed a complete small bowel obstruction in all patients who failed the conservative treatment, and a small bowel resection was necessary in 19 patients (10.0%): among them, only 5 had intestinal ischemia.

**Conclusions:**

Our protocol is safe, and it is a valuable strategy in order to accelerate the decision-making process for management of adhesive SBO, with a percentage of risk of late small bowel resection for ischemia esteemed at 0.9%.

## Introduction

Postoperative adhesive small bowel obstruction (SBO) is a frequent cause of hospital admission, accounting for 16–20% of hospitalizations for acute abdominal pain in a surgical department [1–4]. The Bologna Guidelines for Diagnosis and Management of Adhesive Small Bowel Obstruction, revised in 2018 [5], recommend an initial medical treatment for 72 h before proposing surgical treatment, except when signs of gravity are already present, such as strangulation, peritonitis, and intestinal ischemia. This therapeutic strategy for adhesive SBO remains controversial since a 30–40% risk of failure of nonoperative management has been documented [6, 7]. Many studies have investigated benefits of using oral water-soluble contrast (OWSC) such as diatrizoate meglumine-diatrizoate sodium (Gastrografin^®^, Bayer Schering Pharma) to manage adhesive SBO and predict the need for surgery [8–11]. However, the optimal timing for Gastrografin^®^ administration remains controversial.

In a previous publication in 2009 [12], we conducted a prospective study using Gastrografin^®^ with a 32% rate of delayed surgery after failure of a nonoperative management. We found that Gastrografin^®^ was a useful diagnostic tool in decision-making for surgery, but we were not able to prove that this protocol had a direct therapeutic impact. Based on our previous results, we defined a new protocol to optimize the management of patients with SBO with a fast decision-making process in order to reduce the risk of the need for surgery and length of hospital stay. The aim of this observational study was to evaluate safety and effectiveness of the protocol of a nonoperative management of adhesive SBO with the help of Gastrografin^®^ administration, and to identify risk factors of failure.

## Patients and methods

### Study cohort

From January 2008 to December 2021, we performed a chart review from a database of patients who were diagnosed with adhesive SBO in a single center. According to the Helsinki declaration, an observational study based on a retrospective chart review built exclusively on data, do not need the approval of “committees for the protection of individuals.” All patients were seen on an individual basis preoperatively by the surgeon and provided with verbal and written information.

Patients with hernias, inflammatory bowel disease, suspected or documented history of peritoneal carcinomatosis, pregnancy, or allergy to a component of Gastrografin^®^ were excluded from the study.

An emergency surgery was performed in patients with abdominal tenderness, peritonitis, hemodynamic instability, major acute abdominal pain despite gastric decompression, or CT scan findings of small bowel ischemia (group 1). A laparotomy was usually performed, sometimes a laparoscopic approach, depending on the intestinal distension, the medical history of patient and personal experience of the surgeon.

**Fig. 1:**
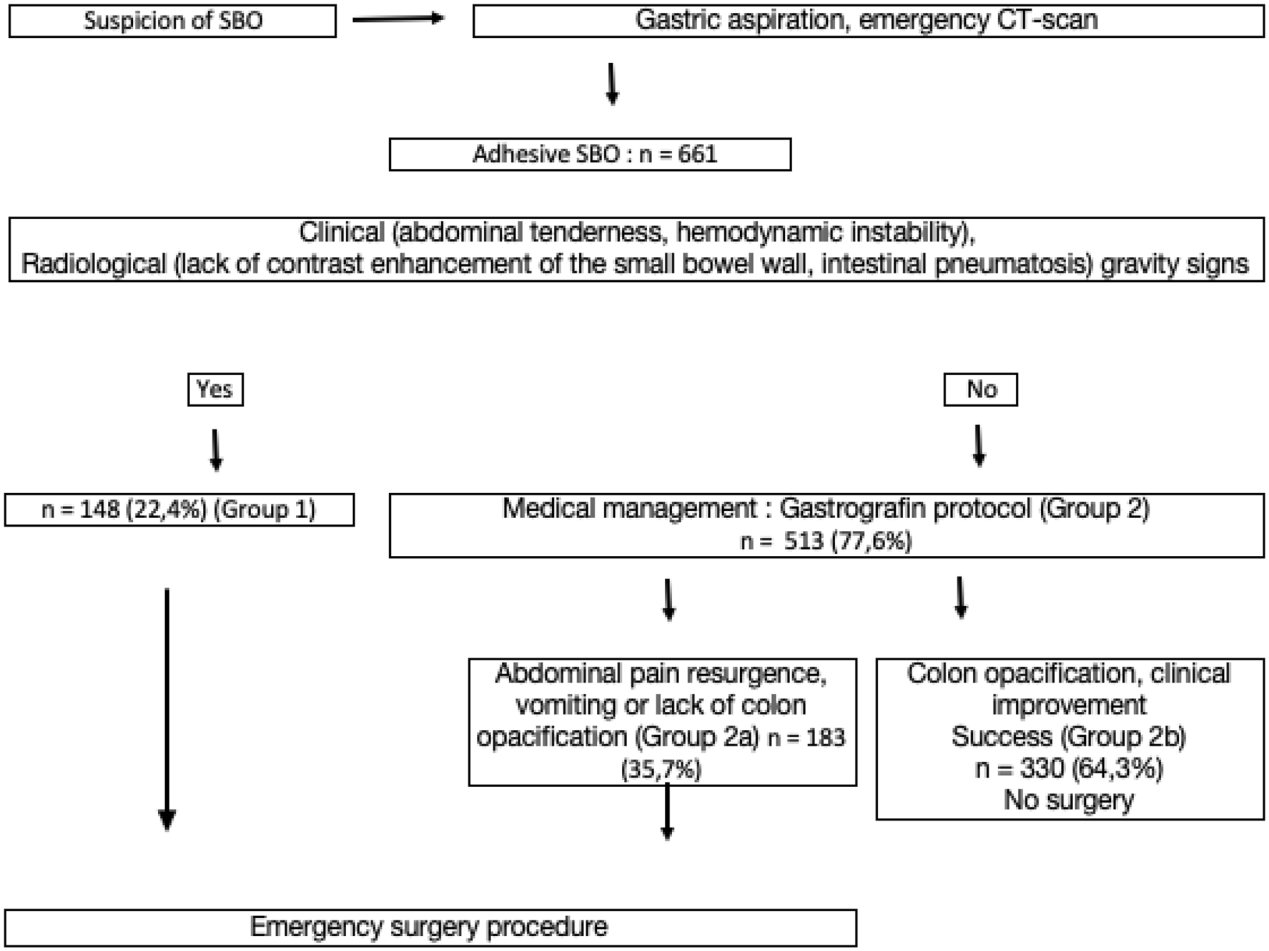
Study management flowchart

Nonoperative management was proposed to patients who did not need emergency surgery (group 2). A gastric decompression with a nasogastric tube was immediately performed in the emergency room. The stomach was aspirated for four hours, then the nasogastric tube was clamped and 100 ml of nondiluted oral Gastrografin^®^ was administered. The nasogastric tube remained clamped for eight hours and an abdominal plain radiograph was taken after that period (8 h after oral Gastrografin^®^ administration). Emergency surgery was performed in patients (group 2a) who had persistent abdominal pain, onset of abdominal tenderness or vomiting during the clamping test, or if the abdominal plain radiograph did not show contrast product in the colon or the rectum. In other cases, the nasogastric tube was removed after 8-h clamping test (group 2b). A progressive refeeding was introduced, starting with liquid diet. Figure 1 presents the flow chart of our study management protocol.

### Statistical analysis 

Results are reported as the median (range) or as the mean ± 1 standard deviation. We used the chi-square test or Student’s *t* test, as appropriate. The first analysis compared group 1 (patients who immediately underwent surgery after admission without Gastrografin® administration) with group 2 (patients managed nonoperatively). A second analysis compared group 2a (patients managed firstly nonoperatively who underwent delayed surgery) with group 2b (patients managed exclusively nonoperatively). The following variables were studied: age, sex, ASA physical status classification, body mass index (BMI), prior abdominal surgery (open or laparoscopic), clinical presentation, blood tests results: serum lactate (mmol/L), white blood cell count (10^9^ cells/L), C-reactive protein level (mg/L), and CT scan features. Statistical significance was defined as *p* < 0.05. All analyses were performed with SPSS statistics software version 13.0.

## Results

A total of 661 patients with adhesive SBO were included in our study (Table 1): 299 males (45%) and 362 females, with a median age of 65 years (range: 19–104 years), and a median BMI of 22 kg/m^2^ (range: 12–53 kg/m^2^). All patients admitted to the emergency room with SBO diagnosis received an abdomen CT scan. CT scan finding of free diffuse peritoneal fluid is present in 66,9% of the operative group (group 1) and in the 35.0% of the conservative group (group 2) (*p* value < 0.001). Radiological findings of bowel devascularization was detected in 33.1% of the operative group, while no one of the patients who underwent the conservative management showed such sign (*p* < 0.001).

**Table 1:** Baseline characteristics of the study population. Comparison between patients who underwent immediate emergency surgery (group 1) and patients who were managed nonoperatively (group 2)

Baseline characteristics	Patients Group 1 (148)	Patients Group 2 (513)	*P* value
Age (years)	66.0 (21–104)	62.2 (19–99)	0.025
Gender (male)	61 (41.2%)	238 (46.3%)	0.265
BMI (kg/m^2^)	23.4 (14–44)	22.8 (12–53)	0.167
Comorbidity	143 (96.2%)	507 (98.8%)	0.064
Abdominal cancer antecedents	35 (23.6%)	132 (25.7%)	0.608
Abdominal radiotherapy antecedents	4 (2.7%)	36 (7.0%)	0.052
Abdominal previous surgery	126 (85.1%)	496 (96.6%)	0.025
Only previous laparoscopy	117 (79.0%)	442 (86.1%)	0.796
Prior SBO	29 (19.5%)	168 (32.7%)	0.002
Prior surgery for SBO	19 (12.8%)	90 (17.5%)	0.174
Abdominal tenderness	56 (37.8%)	10 (1.9%)	< 0.001
Blood test			
White blood cells (10^9^/L)	11,039 (2000–42,000)	10,539 (1300–63,000)	0.325
C-reactive protein (mg/L)	29.1 (1.0–575.0)	20.6 (1.0–424.0)	0.088
Lactates (mmol/L)	1.6 (0.5–22.0)	1.2 (0.5–8.0)	< 0.001
Abdominal CT scan			
Devascularized small bowel	49 (33.1%)	0 (0%)	< 0.001
Peritoneal effusion	99 (66.9%)	180 (35.0%)	< 0.001

Continuous variables are reported as medians, minimum, and maximum values

*BMI* body mass index, *SBO* small bowel obstruction

Patients who underwent immediate emergency surgery (group 1) were older than patients who were managed nonoperatively (group 2): 66.0 years vs. 62.2 (*p* = 0.025). Additionally, patients who experienced a prior SBO were more likely to be managed nonoperatively (*p* = 0.002) (Table 1).

One hundred forty-eight patients (22%) underwent emergency surgery (group 1) because of abdominal tenderness (66 patients, 10%), ischemia of the small bowel on CT scan (51 patients, 7.7%), hemodynamic instability (5 patients, 3.3%), and multiple organ failure (8 patients, 5.4%); among them, 67 patients required small bowel resection (46%) for irreversible ischemia or necrosis. Patients of group 1 had more intense abdominal pain (*p* < 0.001). There was no difference for biologic results (white blood cells count and C reactive protein), except for serum lactates which were higher in patients who needed immediate surgery (*p* < 0.001) (Table 1).

A total of 513 patients (78%) were managed nonoperatively (group 2), including 183 (36%) who finally required surgery (group 2a). Rapidly in front of the persistence of an acute abdominal pain (*n* = 45; 25%) or the occurrence of vomiting after clamping the nasogastric tube (*n* = 68; 37%), or later, after plain radiograph (8 h after the administration of Gastrografin) did not show contrast medium in the colon or the rectum (*n* = 66; 36%). Delayed surgery showed a complete small bowel obstruction in all cases in group 2a, and a small bowel resection was necessary in 19 patients (10%). In those 19 patients, 5 underwent a small bowel resection for intestinal necrosis (2.7%). Among the other 14 patients, intestinal resection was needed in 8 cases for consequent intestinal damage after adhesiolysis, in 3 cases for intestinal stenosis, in 2 cases for tumoral lesions and in 1 case for Meckel’s diverticulum.

The 330 remaining patients (group 2b, 64%) were successfully treated nonoperatively showed a progressive clinical improvement and were discharged after a median delay of 3 days (range: 1–29).

In the second analysis, we compared patients who were successfully treated nonoperatively (group 2b, *n* = 330) with patients who required surgery after the initial attempt at medical management with Gastrografin (group 2a, *n* = 183) (Table 2). We observed significant differences between groups 2a and 2b in the previous SBO (*p* = 0.003): 37.2% of patients who responded to the conservative treatment had already had at least one precedent episode of SBO, versus 24.6% of patients who required delayed surgery.

**Table 2:** Comparison of baseline characteristics between patients in group 2a and patients group 2b

Baseline characteristics	Patients Group 2a (183)	Patients Group 2b (330)	*P* value
Age (years)	60.8 (19–99)	63.1 (19–97)	0.178
Gender (male)	91 (49.7%)	147 (44.5%)	0.260
BMI (kg/m^2^)	22.4 (12.0–37.0)	23.0 (12.0–53.0)	0.189
Comorbidity	181 (98.9%)	326 (98.7%)	0.904
Abdominal previous surgery	168 (91.8%)	301 (91.2%)	0.819
Only previous laparoscopy	157 (85.8%)	285 (86.3%)	0.583
Type of surgery			
Digestive	116 (63.4%)	235 (71.2%)	0.026
Gynecological	47 (25.7%)	87 (26.3%)	0.814
Urological	27 (14.7%)	42 (12.7%)	0.544
Vascular	16 (8.7%)	16 (4.8%)	0.085
Other	1 (0.5%)	1 (0.3%)	0.675
Prior SBO	45 (24.6%)	123 (37.2%)	0.003
Prior surgery for SBO	29 (15.8%)	61 (18.4%)	0.452
Abdominal tenderness	4 (2.2%)	6 (1.8%)	0.773
Blood test			
White blood cells (10^9^/L)	10,365 (1600–301,00)	10,636 (1300–630,00)	0.590
C Reactive protein (mg/L)	21.8 (1.0–355.0)	20.0 (1.0–424.0)	0.669
Lactates (mmol/L)	1.2 (0.5–8.0)	1.2 (0.5–4.6)	0.359
Abdominal CT scan			
Devascularized small bowel	2 (1.0%)	0 (0%)	0.057
Diffuse peritoneal effusion	80 (43.7%)	100 (30.3%)	0.002

We also found a statistically significant difference concerning the presence of moderate to large diffuse peritoneal effusion which was higher in the group of patients who had delayed surgery after failure of conservative treatment: 100 patients (30.3%) in group 2B versus 80 patients (43.7%) in group 2A (*p* = 0.002).

A significative statistic difference was underlined between the group treated conservatively with Gastrografin and the group who underwent immediate surgery concerning inhalation pneumonia, which was lower in the conservative group (0.97% vs 5.40%, *p* < 0.001). In the group of patients who failed conservative treatment we could observe 3 cases of inhalation pneumonia. In our case series, we observed overall 4 deceases presumably due to inhalation pneumonia, 3 of which occurred in the group of patients who required immediate surgery and 1 in the group treated conservatively.

## Discussion

Adhesive SBO is a common cause of emergency department visits and it frequently occurs after abdominal surgery [13]. It remains a relevant clinical issue, in terms of costs and morbidity, with an increase of mortality documented in the elderly population [1, 14]. Most SBO patients can be managed safely with initial nonoperative management [15]. Despite the relevance of the frequency of SBO, the recommended therapeutic strategy remains controversial. An ideal management plan would prioritize immediate operative exploration when signs of gravity are present at admission, ensure early identification of patients who will fail to respond to conservative management, and decrease the number of nontherapeutic abdominal explorations with the use of a water-soluble oral contrast tests. The last edited guidelines for the management of SBO recommend an initial medical treatment for 72 h before proposing surgical treatment, except when signs of gravity are already present [5]. However, delayed surgery seems to be associated with an increased morbidity and mortality rates [16, 17]. The challenge is to find a way to accelerate the identification of those patients who will fail the nonoperative management.

Clinical features and hemato-chemical values can highlight the severity of the SBO, as well as CT findings [18, 19].

In our series, 22% of SBO patients underwent immediate surgery. There were several factors predicting the need for emergency surgery, such as major and persisting abdominal pain, elevated serum lactate levels, and ischemia of the small bowel on CT scan images.

In the systematic review and meta-analysis by Li et al. [20], the pooled sensitivity and specificity of CT for ASBO were 91% and 89%, respectively. Concerning CT findings, in our study, we observed that free peritoneal fluid was present in the 66.9% of the patients who underwent immediate surgery, and in the 35.0% of the patients of the conservative group (*p* < 0.001).

Seventy-eight percent of patients who came in the emergency department for symptoms of adhesive SBO had an initial nonoperative management. Among them, 37% finally needed surgery. This rate is comparable with other case series reporting rates ranging from 20 to 40% [6]. We chose a waiting time of 8 h to decide whether the patient should go to surgery, after the administration of Gastrografin. No evidence in the literature is available for the optimal time to wait before surgery [6].

Concerning the intraoperative findings, of all the 183 patients who underwent deferred surgery, 157 (85.5%) underwent laparotomic adhesiolysis for a single band or matted adherence, while 19 patients had a bowel resection. It is relevant to note that although 10% of patients initially managed nonoperatively had a small bowel resection during delayed surgery, only 2.7% of them had intestinal ischemia. Among those, it is interesting to note that 2 of them suffered from diabetes mellitus and 2 other consulted to emergency room respectively 48 h and 5 days after the onset of the symptoms of intestinal occlusion. The late consulting and the presence of a comorbidity such as diabetes which can camouflage symptoms and signs of gravity may be two conditions of failure of the conservative treatment.

Concerning the prediction of failure of conservative treatment, we found a significant correlation with the peritoneal effusion: 44% of patients with SBO who failed to respond to medical treatment exhibited a moderate or an important intraperitoneal effusion on the CT scan compared with 30% in the group with successful medical treatment. In the group of patients who needed a small bowel resection for ischemia, in 2 cases, we found a major peritoneal effusion on the CT scan. Although abdominal effusion frequently occurs in patients with SBO without being a gravity sign, a significant diffuse peritoneal effusion corresponds to the beginning of small bowel wall suffering, predicting a failure of medical management [13, 21].

Tabchouri et al. [22] published a retrospective study with a series of 154 patients that showed an increase of postoperative mortality and recurrence rate of SBO in patients who failed conservative treatment, with a rate of mortality of 22% in patients who failed medical treatment versus 0% in the group of patients who had emergency surgery. We did not find such results in our study: when comparing patients who had immediate surgery with delayed-surgery patients, the length of the hospital stay was similar (mean: 9 days), and postoperative complications rate seems higher in case of emergency surgery (surgical and medical complications of 14.8% and 34.4% versus 9.3% and 18.0%, respectively).

For patients who were successfully managed nonoperatively, length of hospital stay was way shorter (median of 3 days). This suggests that the use of Gastrografin accelerates return home for patients who do not need surgery and shortens the surgical decision-making time when conservative management fails; in our study, we are unable to prove that Gastrografin exerted a direct therapeutic effect, but it is a useful diagnostic tool for facilitating a rapid decision-making process in the management of patients with SBO.

Although our study was not focused on comparing recurrence rate, we observed in our series that patients managed nonoperatively tended to have a higher risk of subsequent recurrence of SBO: the group managed conservatively had a recurrence rate of 29.9% versus 8.8% of the group treated surgically. This result is coherent with evidence in literature [23]: Meier et al. [24] reported a long-term follow-up of patients treated with surgical versus conservative approach for SBO for a period of 4.7 years, finding a recurrence rate of 29.4% in the conservative group and of 14.0% in the surgical group. In our study, we observed also that patients who had previous SBO were more likely to respond to a conservative treatment (*p* = 0.003).

In our case series, sixty-six patients presenting with SBO had no previous surgery. Among them, twenty-two were operated immediately, while forty-four were treated conservatively. Nonoperative management with Gastrografin was only effective in twenty-nine patients with virgin abdomen (65.9%). In literature, there are few studies reporting the use of oral water-soluble contrast in the management of SBO of virgin abdomen: in the study of Fukami et al., OWSC seems to be equally effective in the management of SBO both in patients with virgin abdomen and patients with previous surgery [25]. Collom et al. showed in his study that the use of OWSC reduced significantly the need for operative intervention in patients with virgin abdomen [26, 27]. Our results show that management of SBO with Gastrografin is feasible in patients with virgin abdomen, without augmenting the risk of deferred surgery.

In conclusion, in our case series we found that a management of adhesive SBO based on clinical and CT scan features is relevant. Immediate surgery was performed in emergency in case of major persisting abdominal pain, hemodynamic instability, and signs of small bowel ischemia on imaging procedures. Besides these conditions, most of the other patients could be managed nonoperatively. Use of Gastrografin after a 4-h period of nasogastric decompression showed to be helpful for management of adhesive SBO and to reduce the duration of the hospitalization stay.

In our study, we included and analyzed a large cohort of 661 patients in a period of 14 years; our protocol was homogenously and rigorously applied by our surgical team in all patients enrolled in the study who underwent an initial medical treatment. The same surgical team was responsible for following the patients along the entire time of the protocol application, assuring the respect of waiting time of 4 h of aspiration by nasogastric tube, dispensation of Gastrografin, and systematic surveillance after Gastrografin administration. Among 513 patients who were treated medically, only 5 (0.9%) needed a small bowel resection for intestinal ischemia.

In conclusion, the aim of this observational study was to evaluate safety and effectiveness of the protocol of a nonoperative management of adhesive SBO with the help of Gastrografin^®^ administration. We find that our protocol is simple, cheap, feasible, and easily reproducible.

## Data Availability

Data is available on request from the corresponding author.
